# Volatile Anesthetic Isoflurane Attenuates Liver Injury in Experimental Polymicrobial Sepsis Model

**DOI:** 10.31480/2330-4871/071

**Published:** 2018-05-22

**Authors:** Sophia Koutsogiannaki, Hui Zha, Koichi Yuki

**Affiliations:** 1 Department of Anesthesiology, Critical Care and Pain Medicine, Boston Children’s Hospital, Boston, MA, USA; 2 Department of Anaesthesia, Harvard Medical School, Boston, MA, USA; 3 Department of Pediatric, Union Hospital, Tongji Medical College, Huazhong University of Science and Technology, Wuhan, China

**Keywords:** Cecal ligation and puncture, Liver, Neutrophil, Leukocyte function-associated antigen-1, Macrophage-1 antigen

## Abstract

Volatile anesthetics are often administered to patients with sepsis for procedural anesthesia or sedation in intensive care units. Sepsis still carries significant morbidities and mortalities, and organ injuries pose major complications. Early liver dysfunction is associated with poor outcome mainly as a result of overwhelming neutrophil recruitment. Leukocyte function-associated antigen-1 (LFA-1) and macrophage-1 antigen (Mac-1) are major adhesion molecules on neutrophils and involved in neutrophil recruitment. We have previously showed that volatile anesthetic isoflurane inhibited LFA-1 and Mac-1. Here we studied the role of isoflurane, LFA-1 and Mac-1 on neutrophil recruitment to the liver and liver injury using experimental polymicrobial abdominal sepsis induced by cecal ligation and puncture (CLP) surgery. We used wild type (WT), LFA-1, Mac-1 and intercellular adhesion molecule-1 (ICAM-1) knockout (KO) mice. Following the induction of sepsis by CLP surgery, a group of mice were exposed to isoflurane for 2 hours. We found that Mac-1 and ICAM-1, but not LFA-1 were involved in neutrophil recruitment to liver. Isoflurane attenuated neutrophil recruitment and liver injury in WT and LFA-1 KO mice. Mac-1 KO mice had limited neutrophil recruitment and liver injury, both of which were not attenuated by isoflurane further, suggesting that isoflurane mitigated liver injury via Mac-1. Mac-1 colocalized with ICAM-1 and fibrinogen on liver tissues. In the presence of fibrinogen Mac-1 bound ICAM-1 significantly more, while LFA-1 bound less to ICAM-1, suggesting that Mac-1 used fibrinogen as a bridging molecule to bind ICAM-1. In conclusion, isoflurane exposure attenuated neutrophil recruitment and liver injury via Mac-1.

## Introduction

Sepsis remains a difficult disease to be dealt with and continues to be a significant health care burden [1]. Organ injury represents a main complication with significant morbidity and mortality, and overwhelming migration of activated neutrophils into organs and subsequent endothelial cell damage are mainly responsible [2]. Patients suffering from sepsis often undergo procedures or are sedated in intensive care unit (ICU) in Europe and Canada under volatile anesthetics [3–6]. A growing literature suggests that volatile anesthetics possess immunomodulatory effects [7,8]. With an increasing interest in using volatile anesthetics beyond operating rooms, particularly for ICU sedation based on their potentially beneficial profiles [9,10], it is clinically important to understand the impact of volatile anesthetics in sepsis and organ injury.

Accumulation of neutrophils is observed in liver during the early development of sepsis [11] and accounts for significant hepatocellular damage, vascular hypoperfusion and ultimately organ dysfunction [12], as supported by attenuated liver injury in experimental sepsis model [13]. While fulminant liver failure is a relatively rare complication of sepsis [14], inflammatory liver damage and hepatic dysfunction can be seen in 34% to 46% of cases [15]. Early hepatic dysfunction in patients with sepsis is a specific and independent risk factor for poor outcome and represents an underappreciated contributor to disease progression and mortality [16]. β2 integrins are considered critical for neutrophil migration through the endothelium [17]. They consist of four members; αLβ2 (CD11a/CD18, leukocyte adhesion-associated antigen-1; LFA-1), αMβ2 (CD11b/CD18, macrophage-1 antigen; Mac-1), αXβ2 (CD11c/CD18) and αDβ2 (CD11d/CD18) [18,19]. LFA-1 and Mac-1 are two major β2 integrins expressed constitutively on neutrophils, and interact with several ligands including a common ligand called intercellular adhesion molecule-1 (ICAM-1), which is constitutively expressed on the endothelial cells and some other cells [20]. We previously showed that commonly used volatile anesthetic isoflurane blocked both LFA-1 and Mac-1 on neutrophils [3,21–24]. Polymicrobial abdominal sepsis model induced by cecal ligation and puncture (CLP) surgery is the model widely used to study sepsis, recapitulating human sepsis [25]. Thus using this model, we studied the mechanism of neutrophil recruitment to the liver and liver injury in sepsis and the impact of volatile anesthetic isoflurane. We found that isoflurane exposure (2 hour) attenuated neutrophil recruitment to the liver and liver injury via its effect on Mac-1, and that Mac-1 and ICAM-1 were involved in neutrophil recruitment likely using fibrinogen as a bridging molecule.

## Materials and Methods

### Mice

All the mice except ICAM-1 KO mice were from Jackson Laboratory (Bar Harbor, ME, USA) and inbred in our animal facilities. ICAM-1 KO mice were kindly given by Dr. Gregory Priebe (Boston Children’s Hospital). CD11a knockout mice (= LFA-1 KO mice) [26], CD11b (= Mac-1) KO mice [27] and ICAM-1 KO mice [28] were previously described. All the mice were on the C57BL/6 background and housed under specific pathogen-free conditions, with 12-hour light and dark cycles. Male mice at 8–10 weeks of age were used for the experiments.

### Cecal ligation and puncture (CLP) model

All the experimental procedures complied with institutional and ARRIVE guidelines [29] regarding the use of animals in research, and were approved by Boston Children’s Hospital animal care and use committee. Polymicrobial abdominal sepsis was induced by CLP surgery, as previously described [3,25]. Briefly, mice were anesthetized with 60 mg/kg ketamine and 5 mg/kg xylazine given intraperitoneally. Following exteriorization, the cecum was ligated at 1.0 cm from its tip and subjected to a single, through and through puncture using an 18-gauge needle. A small amount of fecal material was expelled with gentle pressure to maintain the patency of puncture sites. The cecum was inserted into the abdominal cavity. 0.1 mL/g of warmed saline was administered subcutaneously. Buprenorphine was given subcutaneously to alleviate postoperative surgical pain. Some groups of mice were placed on a nose cone to be continuously exposed to 1% isoflurane using isoflurane vaporizer (VetQuip; New South Wales, Australia) for 2 hours. Isoflurane is often used at the concentration of 1–2% in clinical practice. Mice were euthanized at indicated time points and were subjected to analysis. In some experiments, LFA-1 blocking antibody (M17/4; BioXcell, West Lebanon, NH) 2 mg/kg was given intravenously prior to CLP surgery as we previously described [30].

### Complete blood count and blood chemistry measurement

VetScan HM2 (Abaxis, Union City, CA) was used for complete blood counts. Blood chemistry was performed using Vetscan VS2 (Abxis; Union City, CA, USA).

### Histology and hematoxylin and eosin staining

Mice were anesthetized and underwent transcardiac puncture for perfusion with phosphate-buffered saline (PBS), followed with cold, 4% paraformaldehyde. Tissues were embedded in paraffin wax after graded ethanol and xylene treatment. The tissue blocks were cut into 5-µm sections and mounted on slides for staining. After deparaffinization and rehydration, slides were stained with hematoxylin and eosin, and dehydrated with ethanol and xylene.

### Myeloperoxidase activity assay (MPO assay) of liver

MPO assay was performed as previously described [31]. Briefly, mice were euthanized at 0, 6, 12 and 36 hours after CLP surgery, and the body was flushed with PBS through the pulmonary artery. Liver was removed and immediately snap-frozen and stored at −80 °C until analysis. Frozen liver was thawed, homogenized and resuspended in 50 mM KPO_4_ buffer (pH 6.0) containing 0.5% hexadecyltrimethylammonium bromide and incubated at 60 °C for 2 hours. Following three freeze-thaw cycles, samples were centrifuged and supernatant was subjected to analysis with the addition of o-dianisidine and H_2_O_2_. Absorbance was measured at 450 nm.

### Flow cytometery

Following incubation with Fc-receptor blocking antibody, surface expression of LFA-1 and Mac-1 were probed using M17/4 (anti-CD11a) antibody and N1/70 (anti-CD11b) antibody, respectively. Erythrocytes were lysed using lysis buffer (BD Bioscience). Neutrophil population was gated as anti-Ly6G antibody positive cells.

### Fluorescence immunohistochemistry

ICAM-1, fibrinogen, and Mac-1 expression were probed in liver tissues. Histology sections were deparaffinized as we previously described [30], and probed with fluorescence labeled anti-ICAM-1, fibrinopeptide A and CD11b antibodies.

### Cells

CHO-K1 cells were cultured in HAM-F12 medium/10% fetal bovine serum (FBS). CHO-K1 cells stably transfected with human ICAM-1 and mouse ICAM-1 were made by transfecting pcDNA3.1 plasmids containing human ICAM-1 and mouse ICAM-1, respectively, and selecting with G418. K562 cells stably transfected with Mac-1 were previously described [32] and cultured in RPMI1640 medium/10% FBS and 4 µg/mL puromycin.

### V-bottom well ICAM-1 binding assay with or without fibrinogen

V-bottom well binding assay was performed as we previously described [3]. Briefly, CHO-K1 cells WT, or stably transfected with human ICAM-1 or mouse ICAM-1 were stained with 2’,7’-bis-(2-carboxyethyl)-5-(and 6-)-carboxyfluorescein, acetoxymethyl ester (BCECF-AM) (Life Technologies; Chelmsford, MA, USA). V-bottom wells were coated with 5 µg/mL of human (or mouse) LFA-1 or Mac-1 (R&D Systems; Minneapolis, MN, USA). Some of the wells were then co-incubated with fibrinogen. BCECF-AM stained cells were incubated in V-bottom wells with 1 mM Mg^2+^/Ca^2+^, or 1 mM Mn^2+^/Ca^2+^ at 37 °C for 30 min. Then plates were centrifuged at 200 × *g* for 5 min. Cells that did not bind to plated ligand were accumulated at the center of the well. Fluorescence was read with excitation 485 nm and emission 538 nm. The binding % was defined as [(fluorescence intensity of CHO-K1 WT cell samples)-(fluorescence intensity of CHO-K1 human (or mouse) ICAM-1 stable cell samples)]/(fluorescence intensity of CHO-K1 WT cell samples) × 100 (%).

### V-bottom well Mac-1: Fibrinogen binding assay in the presence of isoflurane

Fibrinogen (1 µg/mL) was coated on V-bottom well. After staining K562 cells stably transfected with Mac-1 using BCECF-AM, they were incubated in V-bottom wells with 1 mM Mg^2+^/Ca^2+^ or 1 mM Mn^2+^ in the presence of isoflurane at various concentrations for 30 min. The rest of procedures were described above.

### Statistical analysis

Data were analyzed as indicated in the corresponding figure legends. Statistical significance was defined as p < 0.05. All the statistical calculations were performed using PRISM 5 software (GraphPad Software; La Jolla, CA, USA).

## Results

### Polymicrobial abdominal sepsis induced neutrophil accumulation into the liver and liver injury

**Figure 1:**
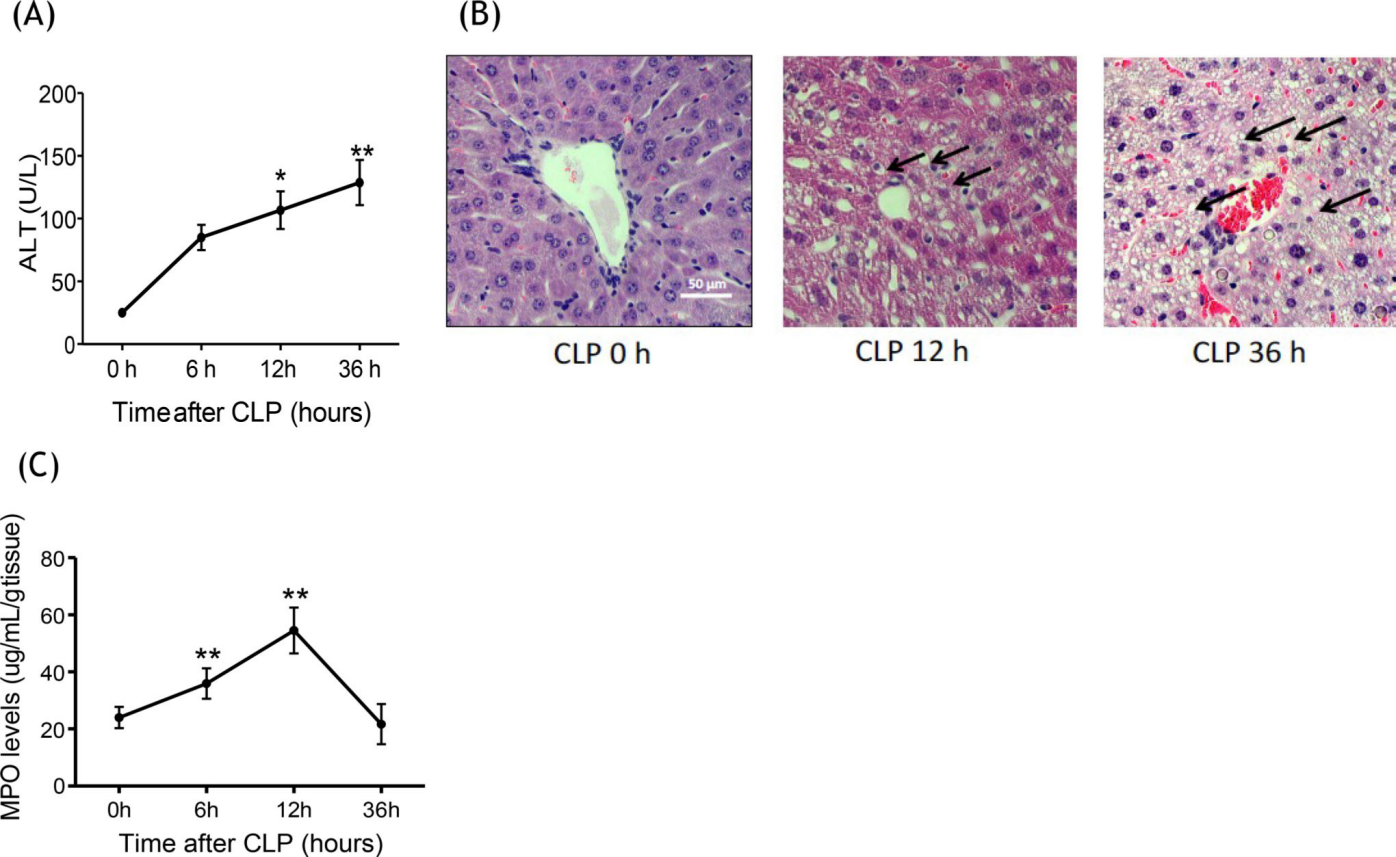
Liver injury and neutrophil recruitment following CLP surgery (A) Liver enzymes ALT were measured at 0, 6, 12 and 36 hours after CLP surgery in WT mice (n = 6–8 per group); (B) Liver histology at 0, 12 and 36 hours after CLP surgery in WT mice. Representative images are shown. Vacuolation was shown in arrow; (C) Myeloperoxidase activity levels of liver at 0, 6, 12 and 36 hours after CLP surgery in WT mice (n = 8 per group). Statistical analysis was performed using one-way ANOVA with Bonferroni post hoc analysis. * and ** denote p < 0.05 and p < 0.01 versus samples at 0 hour, respectively.

The causative role of neutrophils in liver injury in CLP model was previously shown [13]. Here we assessed the time course of neutrophil recruitment to the liver and liver injury using the CLP model in wild-type (WT) mice. Liver injury was demonstrated by the elevation of alanine transaminase (ALT) after CLP over time, which started being significant at 12 hours and progressed till 36 hours after CLP (Figure 1A). Histological analysis also showed hepatocyte swelling and vacuolation along with transmigrated neutrophils at 12 hours after CLP surgery, supporting early stage of liver injury at this time point (Figure 1B) [4,33]. At 36 hours, liver congestion and hepatocyte vacuolation were observed. Next, we examined the time course of neutrophil migration to the liver by measuring the levels of myeloperoxidase (MPO) in liver homogenates. Significant neutrophil accumulation was observed at 6 hours after CLP, and seen most at 12 hours (Figure 1C). Neutrophil recruitment preceded liver enzyme elevation, which further supported neutrophil involvement in liver injury. Since neutrophil accumulation was highest at 12 hours after CLP, we studied the role of β2 integrin at this time point.

### Neutrophil recruitment to the liver was attenuated in Mac-1 KO and ICAM-1 KO mice, but not in LFA-1 KO mice at 12 hours after CLP surgery

**Figure 2:**
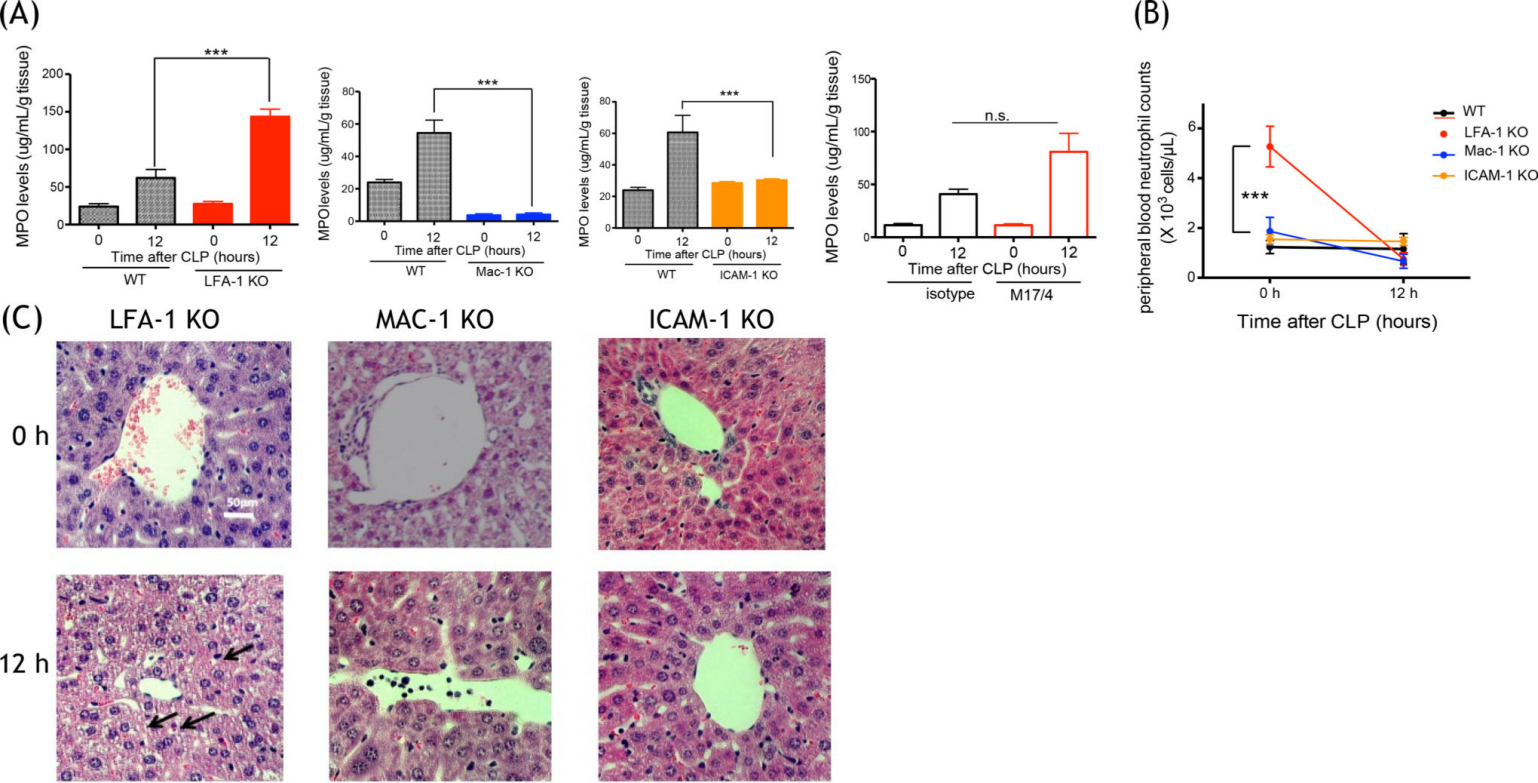
Liver injury and neutrophil recruitment following CLP surgery in LFA-1, Mac-1 and ICAM-1 KO mice (A) Myeloperoxidase activity levels of liver from WT, LFA-1, Mac-1 and ICAM-1 KO mice, WT with or without M17/4 antibody injection (n = 6–8 per group) at 0 and 12 hours after CLP surgery. Statistical analysis was performed using one-way ANOVA with Bonferroni post hoc analysis. *** denote p < 0.001, respectively; n.s. = not significant.; B) Peripheral blood neutrophil counts at 0 and 12 hours after CLP (n = 6 per group). Two-way ANOVA with Bonferroni post hoc analysis. ***denotes p < 0.001 vs. WT; (C) Liver histology at 0 and 12 hours after CLP surgery in LFA-1, Mac-1 and ICAM-1 KO mice. Representative images are shown. Vacuolation was shown in arrow.

MPO levels were significantly attenuated in Mac-1 KO and ICAM-1 KO mice, but not in LFA-1 KO mice at 12 hours after CLP (Figure 2A). The result of LFA-1 KO mice here was compatible with the result of our previous liver MPO staining [30]. Peripheral blood neutrophil counts were compared among all the mouse strains at 0 h and 12 hours after CLP. At 0 hour, LFA-1 KO mice showed significantly higher neutrophil counts than the rest of mouse strains (Figure 2B). At 12 hours, there was no difference in neutrophil counts among different strains. Because that fact that LFA-1 KO mice had higher neutrophil counts at the baseline might complicate our interpretation of neutrophil recruitment to the liver, we also performed LFA-1 neutralization experiment using blocking antibody M17/4 in WT mice. The result showed that blocking LFA-1 did not attenuate neutrophil recruitment to the liver, in line with the result of LFA-1 KO mice experiment (Figure 2A). Liver was less damaged at 12 hours after CLP in Mac-1 KO and ICAM-1 KO mice, while significant vacuolation was observed in LFA-1 KO mice (Figure 2C). Of note, the liver in Mac-1 KO mice showed sinusoid dilation without CLP mice.

### Isoflurane exposure attenuated liver injury and neutrophil recruitment to the liver

**Figure 3:**
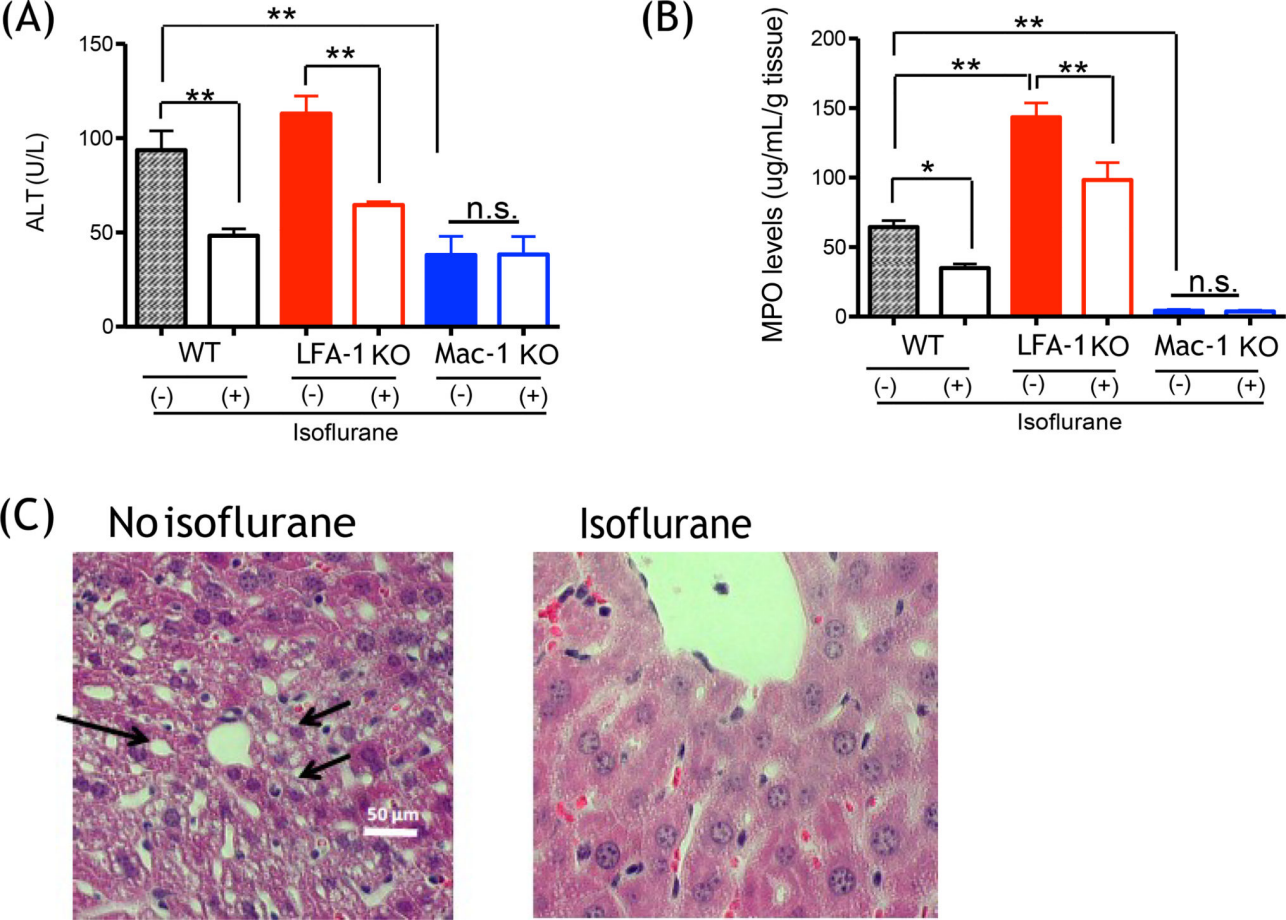
The effect of isoflurane on liver injury and neutrophil recruitment following CLP surgery (A) Liver enzymes ALT were measured at 0 and 12 hours after CLP surgery in WT, CD11a and CD11b mice with or without 1% isoflurane exposure (n = 8 per group); (B) Myeloperoxidase activity levels of liver at 0 and 12 hours after CLP surgery in WT, CD11a and CD11b mice with or without isoflurane exposure (n = 8 per group). Statistical analysis was performed using one-way ANOVA with Bonferroni post hoc analysis. * and ** denotes p < 0.05 and p < 0.01 versus samples at 0 hour, respectively; n.s. = not significant; (C) Liver histology at 12 hours after CLP surgery in WT mice with or without isoflurane exposure. Representative images are shown. Vacuolation was shown in arrow.

We exposed a group of mice to 1% isoflurane for 2 hours after CLP surgery and assessed liver injury and neutrophil recruitment at 12 hours after CLP. Isoflurane attenuated the elevation of ALT and recruitment of neutrophils in WT mice (Figure 3A and Figure 3B). ALT level of LFA-1 KO mice was comparable to that of WT mice, and isoflurane exposure attenuated ALT level and neutrophil levels in liver in LFA-1 KO mice as well (Figure 3A and Figure 3B). In contrast, Mac-1 KO mice showed lower ALT values and neutrophil levels than WT and LFA-1 KO mice at 12 hours after CLP in isoflurane non-exposure experiments, and isoflurane exposure did not affect them, suggesting that the reduction in ALT and neutrophil recruitment by isoflurane in WT mice occurred via its impact on Mac-1 function. We previously showed that isoflurane bound to and inhibited Mac-1 *in vitro* [21], compatible with our *in vivo* finding here. In line with the previous results, isoflurane exposure group showed less injury in liver on histology, demonstrated as less hepatocyte vacuolation (Figure 3C).

### Mac-1 expression increased but LFA-1 expression on neutrophils decreased after CLP surgery and isoflurane attenuated Mac-1 expression

**Figure 4:**
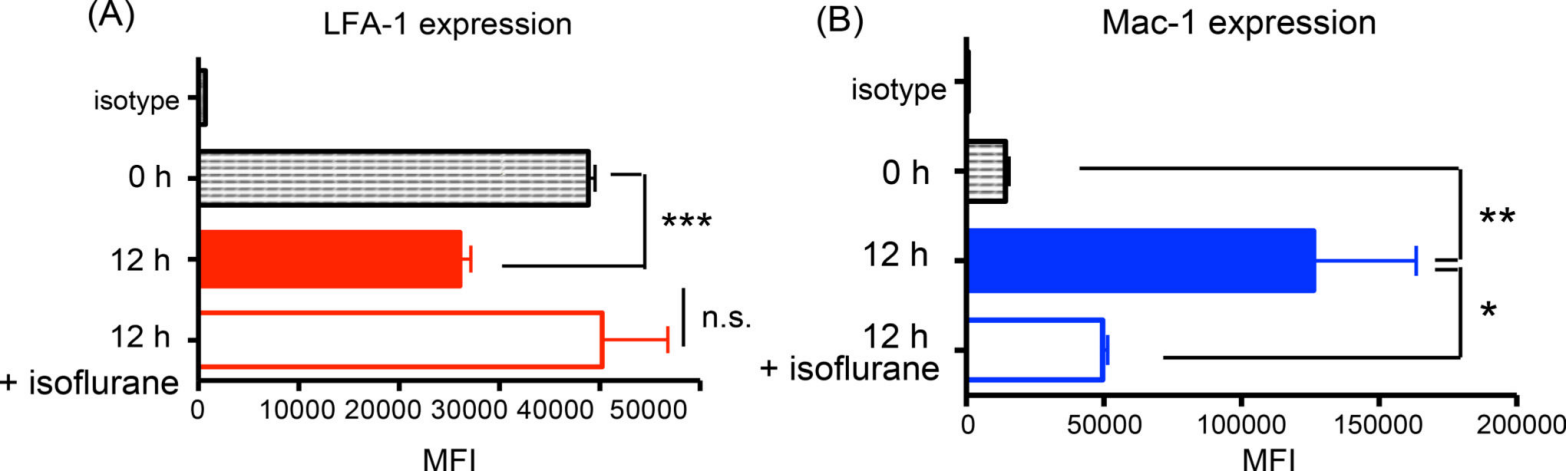
LFA-1 and Mac-1 expression on neutrophils and the expression of their ligands in liver The mean fluorescence intensities (MFIs) of (A) LFA-1 and (B) Mac-1 on peripheral blood neutrophils in WT mice (n = 4–8) at 0 and 12 hours after CLP surgery with or without isoflurane exposure. Statistical analysis was performed using one-way ANOVA with Bonferroni post hoc analysis. *, ** and *** denote p < 0.05, p < 0.01 and p < 0.001 versus time 0 hour, respectively; n.s. = not significant.

LFA-1 and Mac-1 both bind to ICAM-1, but our result suggested that Mac-1 and ICAM-1, but not LFA-1 were involved in neutrophil recruitment to the liver in CLP model. Thus we tested the expression profile of LFA-1 and Mac-1 on neutrophils. On neutrophils LFA-1 expression was reduced but Mac-1 expression increased at 12 hours after CLP (Figure 4A and Figure 4B). Isoflurane exposure did not affect LFA-1 expression, but lowered Mac-1 expression at 12 hours (Figure 4A and Figure 4B). Thus isoflurane could affect Mac-1 function also by lowering its expression level as well in addition to its direct interaction [21]. LFA-1 was still highly expressed on neutrophils at 12 hours after CLP, suggesting that there may be a mechanism that would hinder LFA-1 binding to ICAM-1 in liver. The presence of a bridging molecule between Mac-1 and ICAM-1 was considered as a possibility.

### Mac-1 bound to ICAM-1 more in the presence of fibrinogen and LFA-1 bound less

**Figure 5:**
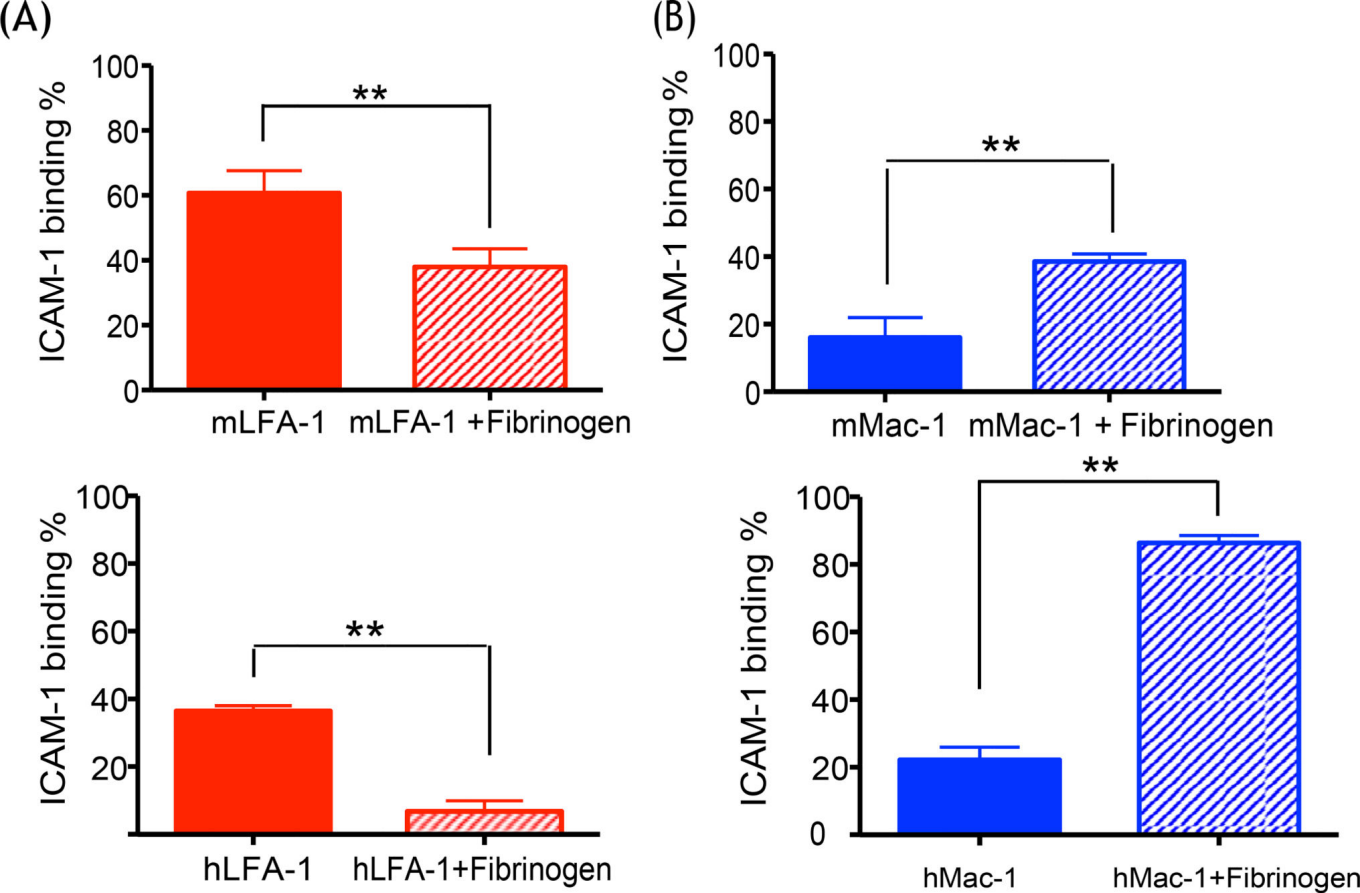
The effect of fibrinogen on LFA-1 and Mac-1 binding to ICAM-1 The binding of LFA-1 and Mac-1 to ICAM-1 was tested *in vitro* with or without fibrinogen (A) Mouse and human LFA-1; (B) Mouse and human Mac-1 were tested. Student t-test was performed. Representative data of three independent experiments (n = 4 per group) are shown. * and ** denotes p < 0.05 and p < 0.01, respectively.

ICAM-1 consists of five extracellular immunoglobulin-like domains (named D1, D2, D3, D4 and D5) [7]. LFA-1 binds to ICAM-1 at D1, while Mac-1 binds at D3 [34]. Fibrinogen is produced by liver [35], and circulates in plasma. It increases its plasma concentration in response to infection and inflammation. It serves as a ligand for Mac-1 but not for LFA-1 [36], and also binds to ICAM-1 at D1 domain, similar to LFA-1, although at distinct sites [37]. Thus, we hypothesized that the binding of fibrinogen to ICAM-1 at D1 would sterically hinder the binding of LFA-1 to ICAM-1. Since fibrinogen binds to both Mac-1 and ICAM-1, we hypothesized that it could serve as a bridging molecule for Mac-1: ICAM-1 interactions in our sepsis model, as previously suggested from *in vitro* study [38]. In support of our hypothesis, our *in vitro* binding experiments showed that binding affinity between ICAM-1 and Mac-1 was weaker than between ICAM-1 and LFA-1 (Figure 5A and Figure 5B), as in line with the previous report [39]. In the presence of fibrinogen, however, LFA-1 binding to ICAM-1 decreased (Figure 5A), while Mac-1 binding to ICAM-1 increased (Figure 5B). Both mouse and human proteins were used in order to assess the human relevance of our model as well.

### Fibrinogen increased and co-localized with Mac-1 and ICAM-1 in liver after CLP surgery

**Figure 6:**
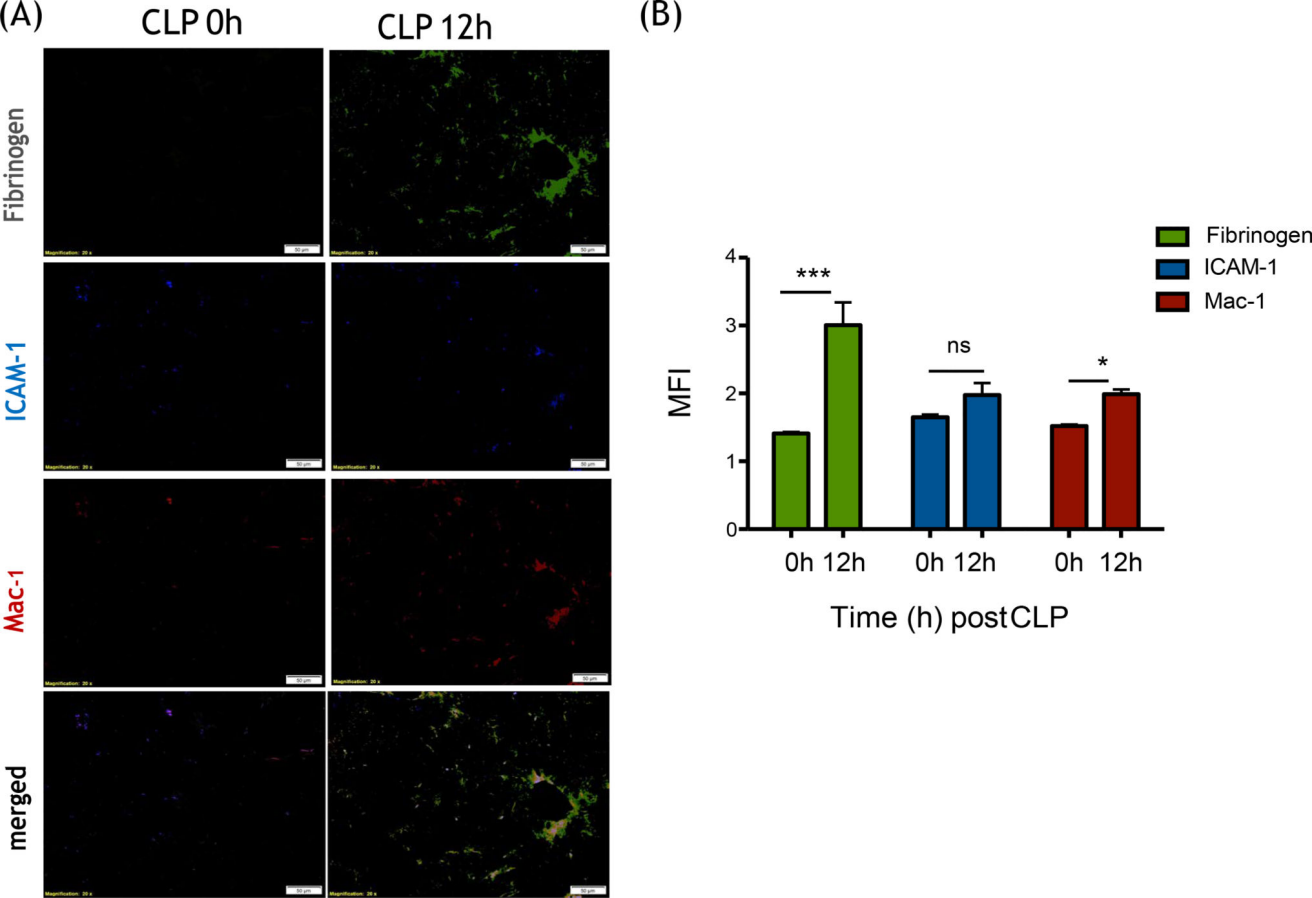
Fibrinogen, ICAM-1 and Mac-1 expression on liver tissues at 0 and 12 hours after CLP surgery (A) Representative immunofluorescence imaging of liver following CLP surgery. Fibrinogen (green), ICAM-1 (blue) and Mac-1 (red) are shown; (B) Fluorescence intensities of fibrinogen, ICAM-1 and Mac-1 were calculated by Image J. Student t-test analysis was performed. * and *** denotes p < 0.05 and p < 0.001, respectively; n.s. = not significant.

Our *in vitro* binding assay suggested that fibrinogen could facilitate the binding of Mac-1 on neutrophils to ICAM-1 on the endothelium, but attenuate the binding of LFA-1. In line with our *in vitro* data, fibrinogen significantly increased in the liver bed at 12 hours after CLP and co-localized with ICAM-1 and Mac-1 *in vivo* (Figure 6A and Figure 6B).

### Isoflurane attenuated Mac-1 binding to fibrinogen

**Figure 7:**
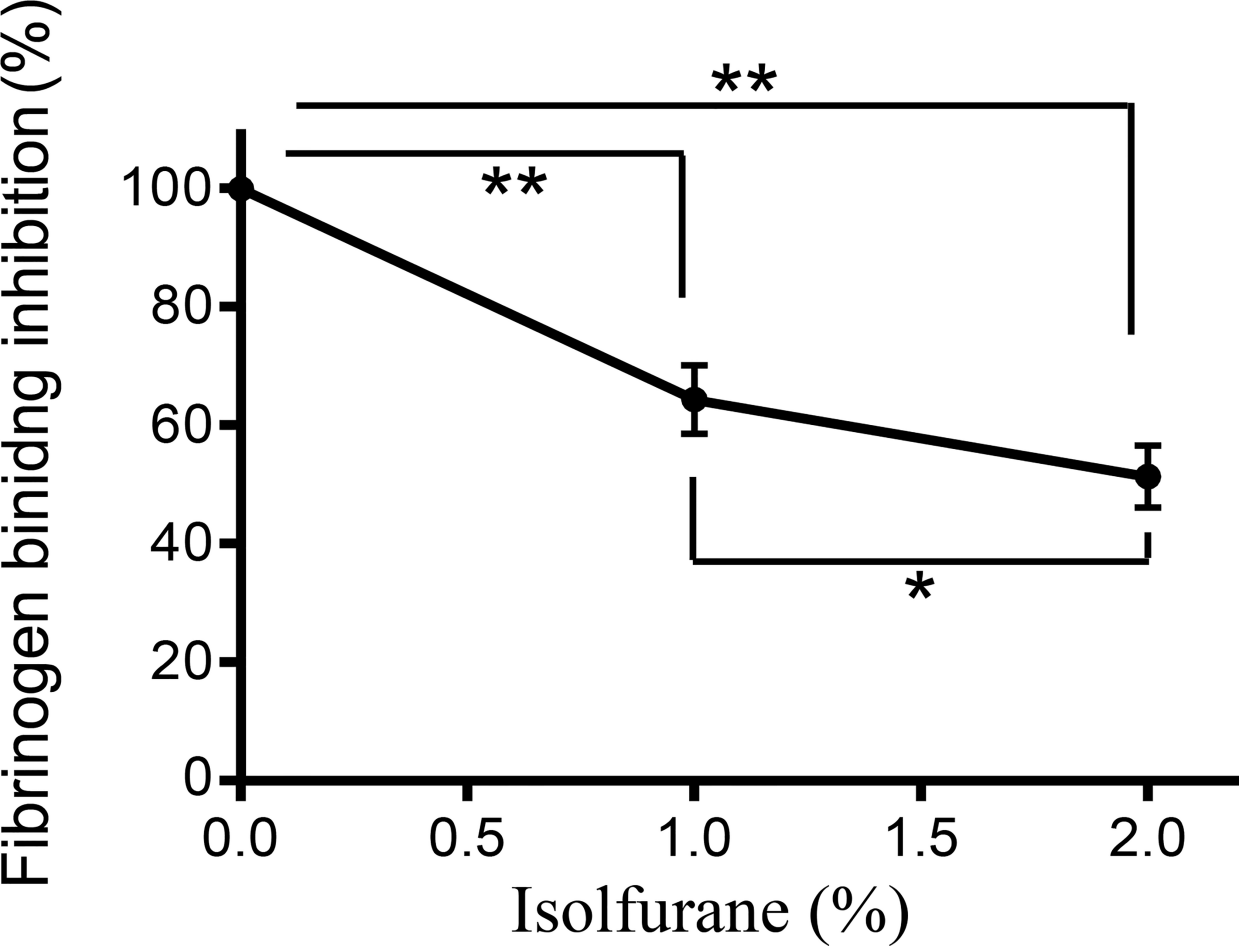
The effect of isoflurane on Mac-1: Fibrinogen binding. The binding of Mac-1 to fibrinogen was tested with or without isoflurane using V-bottom assay. Representative data of three independent experiments (n = 4) were shown. One way ANOVA with Bonferroni post hoc analysis was performed. * and ** denote p < 0.05 and p < 0.01, respectively.

We also tested the binding of Mac-1 to fibrinogen in the presence of isoflurane. We found that Mac-1 binding to fibrinogen was inhibited under isoflurane in a dose-dependent manner (Figure 7). This further supported the idea that isoflurane blocked the interaction of Mac-1 to fibrinogen and attenuated neutrophil recruitment to the liver in our model.

## Discussion

**Figure 8:**
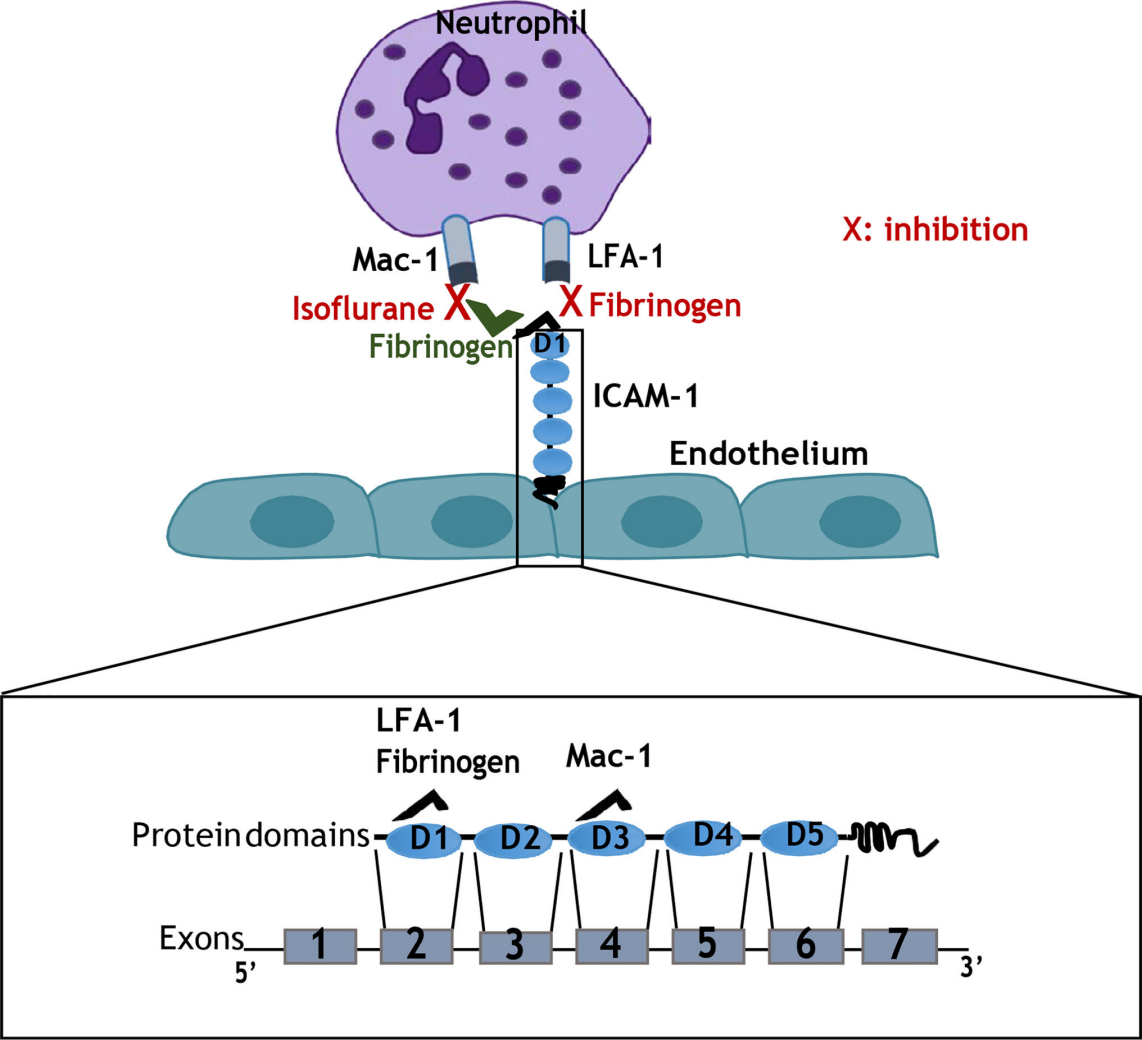
Proposed mechanism of neutrophil recruitment to the liver following sepsis (A) Mac-1 binds to ICAM-1 either directly or indirectly through fibrinogen as a bridge. Fibrinogen acts as an LFA-1 antagonist and block its binding to ICAM-1. Isoflurane blocks Mac-1 function; (B) Schematic representation of mRNA and full length protein of ICAM-1. ICAM-1 binding domains to LFA-1, Mac-1 and fibrinogen are indicated on the scheme.

Here we demonstrated that short (2-hour) isoflurane exposure attenuated liver injury in experimental polymicrobial abdominal sepsis model, Mac-1 and ICAM-1 were involved in neutrophil recruitment to the liver, but LFA-1 was not, and the mechanism of isoflurane-mediated attenuation of liver injury was via its effect on Mac-1 function. Our results also suggested that fibrinogen might contribute to Mac-1: ICAM-1 mediated neutrophil recruitment to the liver. Fibrinogen could favor Mac-1 binding to ICAM-1 by competing with LFA-1 for binding to ICAM-1 on the endothelium, and isoflurane blocked Mac-1 to fibrinogen binding. The suggested mechanism is illustrated in Figure 8.

β2 integrins on the neutrophils are known to contribute to the pathogenesis of a variety of diseases [40]. β2 integrins are transmembrane surface receptors that function as adhesion molecules, and play a critical role in neutrophil migration [17], and we previously showed that isoflurane directly bound to and inhibited LFA-1 and Mac-1 and affected neutrophil functions *in vitro* and *in vivo* [3,21–24]. A growing literature suggests the importance of β2 integrins in the intravascular and transendothelial migration of neutrophils during inflammation and sepsis [14], and it was not surprising that isoflurane exposure reduced neutrophil recruitment to the liver in our model. We demonstrated that in early stage of liver injury during CLP sepsis, neutrophils were recruited to the liver using Mac-1, but not LFA-1 on their surface. Previously, we demonstrated that neutrophil recruitment to the peritoneal cavity was dependent on LFA-1, not Mac-1 in the CLP model [3]. These results indicate that neutrophil recruitment to different organs and tissues might be governed by different mechanisms. This notion is in accordance with previous studies suggesting tissue-specific neutrophil recruitment during inflammation [41]. The specificity observed is attributed to the unique cell populations and structures of some organs like lungs, kidneys and liver. Liver has been extensively described as a unique environment for leukocyte recruitment [42]. Fibrinogen could act as a bridging molecule between Mac-1 on neutrophils and ICAM-1 on the endothelium as well, facilitating neutrophil adhesion and transmigration, as has been previously suggested [38,43]. Fibrinogen may sterically hinder LFA1:ICAM-1 interactions by bridging Mac-1:ICAM-1 interaction.

The attenuation of liver injury by isoflurane exposure has been previously reported in *in vivo* animal models of ischemia-reperfusion injury and clinical setting [4,44–46]. Neutrophils are main players in ischemia-reperfusion injury [47], and Mac-1 on neutrophils is responsible for this process [48,49]. Clinical study was done in patients who underwent liver resection using Pringle’s maneuver (repeated ischemia and reperfusion) under isoflurane or propofol anesthesia, and patients assigned to isoflurane arm showed less liver injury based on liver function test [44]. The impact of isoflurane on neutrophils in ischemia-reperfusion injury has not been examined in these studies [4,46], but this is expected because isoflurane blocks Mac-1 [21]. Here we examined the effect of isoflurane on neutrophil recruitment to liver in experimental polymicrobial abdominal sepsis model, as neutrophils were involved in sepsis-induced liver injury [13,50]. We showed that 2-hour of isoflurane exposure attenuated neutrophil recruitment to the liver and liver injury via Mac-1. The difference between our model and the previous ischemia-reperfusion models is that our model involved infection, while the ischemia-reperfusion injury models were sterile. In fact, we previously showed that prolonged (6-hour) isoflurane exposure worsened systemic bacterial loads by attenuating neutrophil recruitment to the peritoneal cavity and phagocytosis and did not protect liver from injury, while just 2-hour exposure did not affect bacterial loads [3]. 2-hour suppression of immune function by isoflurane would not have been enough to change bacterial loads, but 6-hour exposure would have been long enough to suppress immune function and affect bacterial loads given a short doubling time of bacteria. Thus isoflurane can act as a double-edge sword. It can provide tissue protection, but can also worsen disease process depending on the type of disease and duration of exposure. It is imperative to consider the context when discussing the benefit of isoflurane exposure. This is particularly important given that isoflurane may be a choice of sedation in ICU [5,6]. Additional consideration should be taken that the responsible molecule of neutrophil recruitment to the liver may differ in different models (thus, different diseases), so that we may not be able to expect similar response from isoflurane. For example, neutrophil recruitment to the liver has been studied in lipopolysaccharide (LPS) induced sepsis model [14,51]. In this model, CD44, not Mac-1 was considered to be responsible for neutrophil recruitment [51]. Elevation of interleukin (IL)-10 downregulates the expression of Mac-1 and CD44 instead becomes dominant in this model [52]. We have previously shown that blood IL-10 levels were not statistically significant between 0 and 12 hours post-CLP [30]. In addition, Mac-1 expression was rather enhanced at 12 hours after CLP. CLP model is considered to use a different mechanism to elicit inflammatory responses than LPS model [53]. Notably isoflurane was not protective from liver injury in LPS induced liver injury model [54].

Considering the tissue specific neutrophil recruitment pattern, Mac-1 and fibrinogen may be as an attractive option to attenuate liver injury in polymicrobial sepsis and organ ischemia-reperfusion. However, as indicated by the different results of 2-hour and 6-hour isoflurane exposure, targeting Mac-1 itself to attenuate neutrophil recruitment to liver for an extended period of time is not likely to be a practical approach in sepsis, and we need to consider a duration of inhibition. Mac-1 serves as a complement receptor. Mac-1 binds to iC3b and plays a significant role in complement-mediated phagocytosis and it has been previously shown that deficiency of Mac-1 significantly impairs bacterial phagocytosis in sepsis [3]. Developing and using an ultra-short acting drug to inhibit Mac-1 for a short duration may be a consideration. Targeting fibrinogen may be another consideration. However, blocking the binding of fibrinogen to D1 of ICAM-1 may also allow LFA-1 mediated neutrophil recruitment, since they bind to distinct areas on D1 domain [43]. In addition, fibrinogens are suggested to bind to Mac-1 at multiple sites [36]. Fibrinogen is a protein with two pairs of polypeptides consisting of α, β and γ chains. Binding sites of fibrinogen to Mac-1 are located in the C-terminus of α, β and γ chains [36]. The binding site of the C-terminus of γ chain (N^390^RLSIGE^396^) was tested in transgenic mice [55]. However, mice containing γ chain mutant (A^390^RLSIGA^396^) demonstrated the defect of *S. aureus* clearance *in vivo*. Neutrophils from the mice showed attenuated binding to Mac-1 [55]. Thus, simple blocking the interaction between Mac-1 and fibrinogen for an extended period may not help in the setting of infection either. Identifying the binding sites of fibrinogen with Mac-1 in liver and in infected tissues, if they differ could possibly allow us to perform selective binding inhibition in different tissues.

In summary, we demonstrated that short isoflurane exposure attenuated neutrophil recruitment and liver injury in experimental polymicrobial sepsis model via Mac-1, and we suggested that fibrinogen favored Mac-1:ICAM-1 interaction by acting as a bridging molecule. We showed isoflurane blocked the binding of Mac-1 to fibrinogen. Given the growing body of evidence supports that anesthetics possess immumomodulatory effects, it is critical to understand their functional effects and the underlying mechanism so that this knowledge could be reflected in clinical practice. In addition, identifying and characterizing the molecular mechanisms of neutrophil recruitment within the liver during sepsis may reveal novel therapeutic strategies to prevent immune-mediated organ dysfunction during severe sepsis.
